# The value of promoting “Internet Plus” home-based pulmonary rehabilitation for university staff with chronic obstructive pulmonary disease

**DOI:** 10.3389/fpubh.2026.1976082

**Published:** 2026-09-30

**Authors:** Jia Wei, Haijun Zhang, Yiwei Wang, Qiaoli Su

**Affiliations:** 1 Department of Integrated Care Management Center, Wangjiang Hospital, Sichuan University, Chengdu, Sichuan, China; 2 Department of General Inpatient, Wangjiang Hospital, Sichuan University, Chengdu, Sichuan, China; 3 Department of General Practice, West China Hospital, Sichuan University, Chengdu, Sichuan, China

**Keywords:** “Internet Plus” technology, chronic obstructive pulmonary disease, home-based rehabilitation, pulmonary rehabilitation, telerehabilitation, university staff

## Abstract

Pulmonary rehabilitation (PR) is a non-pharmacological intervention for chronic obstructive pulmonary disease (COPD). “Internet-Plus” technologies create opportunities for home-delivered PR to overcome barriers of center-based rehabilitation, yet real-world implementation evidence among university staff with COPD remains scarce. This mini review synthesizes six major “Internet-Plus” home-based PR modalities: short message service (SMS)/telephone follow-up, social media platforms, video conferencing telerehabilitation, wearable devices and Internet of things (IoT) systems, smartphone applications and virtual-reality-integrated rehabilitation. Available evidence indicates that telehealth PR yields comparable clinical outcomes to center PR in many studies, though considerable heterogeneity across trials leads to inconsistent conclusions regarding exercise capacity, quality of life and hospitalization endpoints. Notably, no interventional studies have delivered telehealth PR specifically for university staff with COPD. We conceptually propose that university staff with high digital literacy and structured occupational settings, constitute a promising target population for translational research in this domain. The review concludes with a conceptual framework for designing precise, intelligent home PR management within university communities.

## 1 Introduction

Chronic obstructive pulmonary disease (COPD) is a prevalent chronic respiratory disorder characterized by progressive irreversible airflow limitation, bringing heavy family-social burdens and impaired quality of life (1). The 2018 China Pulmonary Health Survey (CPH) reported a 13.7% COPD prevalence among Chinese adults ≥40 years old (2). Separately, the prevalence of COPD among well-educated people aged over 50 years was approximately 4.8–4.9% (3). Pulmonary rehabilitation (PR), a key non-pharmacological therapy for COPD, relieves dyspnea and improves exercise capacity (4). Nevertheless, conventional hospital-based PR suffers from low acceptance, poor adherence and insufficient coverage (5–7). With the continuous development of “Internet Plus” technology, its integration with healthcare has created new possibilities for home-based PR among COPD patients. The Global Initiative for Chronic Obstructive Lung Disease (GOLD) guideline recommends that patients with COPD may choose either center-based or telehealth rehabilitation, as both modalities demonstrate comparable clinical outcomes (1). While guideline strongly recommends PR for stable COPD patients, heterogeneity in telehealth intervention designs across studies prevents identification of a superior telehealth PR model (8). Currently, PR is mainly delivered in secondary and tertiary hospitals, and limited research data are available from university-based functional communities. To date, no empirical studies have evaluated “Internet Plus” home-based PR specifically among university staff with COPD. While university personnel have relatively high educational attainment and health awareness, they are theoretically suitable targets for the implementation of “Internet Plus” home-based PR (9). We therefore propose this population as a conceptually promising target for translational implementation of remote PR. This mini review summarizes the current application of “Internet Plus” technology in home-based PR for COPD and discusses the conceptual feasibility of promoting this model within university communities, to inform optimized intelligent home-based PR management for university staff. A systematic literature search was performed in PubMed, CNKI and Wanfang for studies published from January 2015 to August 2026. Full reproducible search strings, inclusion/exclusion criteria and screening record counts are available in Supplementary materials.

## 2 Application models and efficacy of “Internet Plus” technology in home-based pulmonary rehabilitation for COPD

“Internet Plus” technology integrates cloud computing, big data analytics, and the Internet of Things (IoT) to extend services across diverse professional domains; it is centered on internet-based platforms and characterized by convenience, efficiency, and personalization (10). Widespread mobile internet, medical apps and wearable devices facilitate mHealth, advancing home chronic disease care (11). PR, a comprehensive intervention combining exercise, education and behavioral change for chronic respiratory diseases, aims to enhance patients’ physical-mental health (12). Its uptake remains low in China (24.69%) for long-term COPD management (13). Remote PR yields similar safety and efficacy as traditional PR with better adherence (14). “Internet Plus” home-based PR overcomes geographic limitations and supports multiple remote services for COPD, though optimal implementation and platform standards are still lacking. In this section, we outline six common modalities and synthesize the relevant literature. The included primary studies and meta-analyses are detailed in Table 1.

**Table 1:** COPD Internet Plus home-based pulmonary rehabilitation modalities and GRADE evidence certainty.

Application models	First author/year	Modality	Country	Sample size	Population	Intervention duration	Comparator	Key outcomes	Evidence certainty	Study type
Short message service and telephone follow-up	Kasim 2025 (15)	Telephone interviews	India	62	GOLD stages 1–3 stable COPD	12 weeks	None	Pulmonary function; disability status	Low	Prospective, single-arm, pre–post interventional study
	Jiang 2016 (16)	Messages	China	102	Stable COPD	24 weeks	Group 1, participant-initiated weekly scheduled calls to researchers. Group 2, researcher-initiated weekly calls to participants. Group 3, daily group SMS; participants/caregivers reply via SMS.	Self-efficacy (Ex-SERS); dyspnea (mMRC); health status (CAT)	Moderate	3-arm parallel RCT
	Redfern 2024 (17)	Messages and telephone	Australia	316	Cardiovascular disease or chronic respiratory disease	6 months	Automated text message program vs. usual care (no message program)	Exercise capacity (6MWD); BMI; smoking status; physical activity; diet	High	Multicentre, single-blind randomized controlled trial
Social media platforms	Li 2022 (18)	WeChat	China	150	Stable COPD	12 months	Group A, PR maintenance at home via WeChat. Group B, PR maintenance at hospital. Group C, usual care.	COPD exacerbations; exacerbation-related hospitalizations; ED visits	High	Single-center randomized Controlled Trial
	Yao 2021 (19)	WeChat	China	101	Post-discharge COPD exacerbation patients	8 weeks	WeChat small program vs. usual care	Pulmonary function (FEV1/FVC); dyspnea (mMRC); exercise capacity (6MWD); BODE index; health status (CAT); arterial blood gas	Low	Prospective non-randomized controlled interventional study
	Zheng 2026 (20)	WeChat	China	174	Post-discharge COPD exacerbation patients	6 months	WeChat-based hospital-community-family integrated care model vs. traditional care model	Pulmonary function; health status (CAT); exercise capacity (6MWD); cost evaluation	Low	Retrospective Cohort Study
Video conferencing	Bhhat 2022 (21)	Video PR intervention	United States	128	Adults with COPD	12 weeks	Center-based PR vs. telehealth PR intervention	Exercise capacity (6MWD); dyspnea	Low	Retrospective Cohort Study
	Knox 2019 (22)	Live video conferencing	United Kingdom	45	Moderate to severe COPD	7 weeks	Live video conferencing vs. PR in hospital	Adverse events; early clinical changes	Low	Prospective Cohort Study
	Dai 2024 (23)	Video conference-based telerehabilitation	Not applicable	3,030	Chronic respiratory disease	8 weeks to 2 years	Telerehabilitation vs. control usual care	Exercise capacity (6MWD); QoL (SGRQ); dyspnea (mMRC); health status (CAT); anxiety/depression (HADS); pulmonary function	High	Systematic review and meta-analysis
	Bhatt 2019 (24)	Video telehealth PR intervention	United States	240	Post-discharge COPD exacerbation patients	12 weeks	Video telehealth PR intervention vs. usual care	30-day all-cause readmission; exacerbation-related readmission; time to first readmission	Low	Retrospective Cohort Study
Wearable devices and IoT systems	Zhang 2025 (25)	Wearable devices	China	55	Moderate stable COPD	10 weeks	Home-based PR (wearable) vs. outpatient PR	Pulmonary function; CPET; symptoms and HRQoL (SGRQ, CAT, mMRC)	Moderate	Single-center randomized Controlled Trial
	Ward 2021 (26)	Activity monitor	United Kingdom	19	Stable COPD	6 weeks	none	Exercise adherence (walking speed/cadence); device usage; physical activity	Very low	Single-arm feasibility study
	Wu 2024 (27)	IoT systems	China	86	GOLD stage 3 stable COPD	4 weeks	The Internet of Things PR vs. usual care	Pulmonary function; length of hospital stay; duration of NIV use; HRQoL; treatment adherence; patient satisfaction	Moderate	Single-center randomized Controlled Trial
	Zhuang 2025 (28)	Web-based remote digital health management system	Not applicable	2027	COPD	Less than 3 weeks to more than 2 years	Control group	HRQoL (CAT, SGRQ); self-efficacy; functional capacity (6MWD, mMRC); healthcare use (hospital and ED visits)	High	Systematic review and meta-analysis
Mobile applications	Gloeckl 2025 (30)	Mobile app	Germany and Switzerland	278	COPD, age ≥40 years	12 weeks	Control group (no access to the app)	Health status (CAT); exercise capacity (1MSTST); anxiety/depression (HADS); HRQoL (VR-12); patient activation (PAM-13); healthcare resource use; adverse events; app satisfaction	High	Multicentre, randomized controlled trial
	Spielmanns 2023 (31)	Kaia COPD app	Germany and Switzerland	60	GOLD stage 2–4 COPD	6 months	Control group	Physical activity (daily steps); health status (CAT); QoL (CRQ); exercise capacity (1MSTST)	High	Multicentre, randomized controlled trial
	Chung 2024 (32)	Smartphone app	Not applicable	1,050	Adults with COPD	21 days to 6 months	Conventional pulmonary rehabilitation, including exercise programs (center-based rehabilitation or education)	Exercise capacity (6MWD); health status (CAT); dyspnea (mMRC); QoL (SGRQ); exacerbation-related hospitalization	High	Systematic review and meta-analysis
Virtual Reality	Chai 2023 (33)	Virtual reality	Not applicable	751	Adults with COPD	2 weeks to 24 weeks	Pulmonary rehabilitation therapy alone	Pulmonary function; exercise capacity (6MWD); depression; anxiety	High	Systematic review and meta-analysis
	Qi 2025 (35)	Virtual reality	Not applicable	809	Adults with COPD	2 weeks to 16 weeks	Traditional therapy alone	Pulmonary function; exercise capacity (6MWD); depression; anxiety; QoL; adherence; patient satisfaction	High	Systematic review and meta-analysis
	Chen 2025 (36)	Virtual reality	Not applicable	1,052	Adults with COPD	Minimum duration of 2 weeks	Usual care or PR without VR	Pulmonary function; exercise capacity; dyspnea; health status; oxygenation; acceptability and engagement	High	Systematic review and meta-analysis

Evidence certainty was graded using the GRADE approach. 6MWD, 6-min walk distance; 1MSTST, 1-min sit-to-stand test; BODE, body mass index, airflow obstruction, dyspnea, and exercise capacity index; BMI, body mass index; CAT, COPD Assessment Test; COPD, chronic obstructive pulmonary disease; CPET, cardiopulmonary exercise testing; CRQ, Chronic Respiratory Disease Questionnaire; ED, emergency department; Ex-SERS, Exercise Self-Efficacy Scale; FEV1, forced expiratory volume in 1 s; FVC, forced vital capacity; GOLD, Global Initiative for Chronic Obstructive Lung Disease; HADS, Hospital Anxiety and Depression Scale; HRQoL, health-related quality of life; mMRC, modified Medical Research Council dyspnea scale; NIV, non-invasive ventilation; PAM-13, Patient Activation Measure 13; PR, pulmonary rehabilitation; SGRQ, St George’s Respiratory Questionnaire; SMS, short message service; VR-12, Veterans RAND 12-item Health Survey.

### 2.1 Short message service and telephone follow-up

Short message service (SMS) and telephone follow-up, basic forms of “Internet Plus” technology, serve as alternatives to traditional face-to-face follow-up and can strengthen self-management and encourage healthy behaviors. Kasim et al. conducted a prospective, single-arm, pre-post interventional study among stable COPD patients and demonstrated that telephone follow-up management significantly improved lung function and reduced disability rates, with trends toward improved self-management and activity capabilities, although the latter differences did not reach statistical significance (15). Jiang et al. carried out a 3-arm parallel randomized controlled trial (RCT) proving that mobile SMS-supervised PR training enhanced exercise capacity, alleviated dyspnea, and improved quality of life (16). A multicenter RCT among cardiac and pulmonary rehabilitation attendees revealed that Integrated Text Messaging (ITM) improved anxiety and depression plus program attendance (17). The educational and supportive text message program would be suitable for implementation alongside rehabilitation programs. Telephone and SMS management requires little technical proficiency from patients, works reliably regardless of network signal quality, and suits older adults without smartphone operating capabilities. However, SMS messages lack personalization, and telephone follow-up is constrained by language proficiency and comprehension ability; therefore, when used alone, these approaches may yield satisfactory effects.

### 2.2 Social media platforms

Multi-device social media platforms enable communication among medical staff, patients, and caregivers, as exemplified by PatientsLikeMe and WeChat. Via online carriers, social media platforms support health education, consultation, follow-up and dynamic home PR adjustment, improving adherence and peer support. Li et al. conducted a single-center RCT assigning stable COPD patients with completed initial PR to 1-year WeChat-based home PR, hospital-based PR, or conventional PR (18). WeChat-based and hospital-based PR showed comparable clinical improvements in 6-min walk distance (6MWD), COPD Assessment Test (CAT) scores, and modified Medical Research Council Dyspnea Scale (mMRC) scores compared with conventional PR groups, and both were identified as independent predictors of reduced risk of acute exacerbation of COPD (AECOPD). Similarly, Yao et al. implemented WeChat mini-program-based PR in a prospective non-randomized controlled interventional study and showed superior arterial blood gas results, 6MWD, mMRC, and CAT scores versus usual home-based PR in stable COPD (19). Another retrospective study demonstrated that for moderate-to-severe COPD patients post-discharge, the WeChat-based digital hub model not only enhances lung function, quality of life, and exercise capacity, but also drives significant cost savings (20).

### 2.3 Video conferencing

Video conferencing, mobile telephone-based remote supervision, and video transmission can support guidance for home-based exercise training. A case–control study involving COPD patients who could not participate in center-based PR found that real-time interactive remote video PR using a smartphone required minimal equipment and achieved efficacy comparable to center-based PR in alleviating dyspnea symptoms and improving cardiopulmonary function (21). A prospective cohort study conducted by Knox et al. confirmed that video conferencing connecting local sites with standard PR programs was safe and feasible, demonstrating short-term clinical benefits equivalent to center-based PR while reducing staffing requirements and travel expenses (22). A meta-analysis concluded that video conference-based telerehabilitation showed significant improvements in daily activity capacity, respiratory function, and enhancing mental health status in the short-term (≤6 months) observation. However, in long-term follow-up (>6 months) only mMRC and Hospital anxiety and depression scale (HADS) were statistically significant (23). Furthermore, video conferencing-guided home-based PR has been shown to reduce hospital readmission rates and hospitalization frequency (24).

### 2.4 Wearable devices and internet of things systems

Wearable devices, with simple operation, portability and wireless data transmission, use IoT and built-in sensors to collect real-time health data and establish integrated disease management systems, enabling patients to obtain professional home PR and timely medical support free from geographic and hardware limits. A single-center RCT conducted by Zhang et al. found comparable improvements in exercise function, symptoms and quality of life between outpatient PR and wearable-supervised home-based PR for moderate COPD, whereas home-based PR achieved higher patient adherence (25). In a single-arm feasibility study, Sarah Ward et al. demonstrated that wearables raised daily step counts and exercise adherence. Higher step counts correlated with reduced dyspnea and improved emotional status, favoring remote walking prescriptions in PR (26). In a single-center RCT, Wu et al. applied an IoT-enabled system for personalized home PR in GOLD-3 COPD patients (27). Real-time data supported targeted clinical interventions, yielding better lung function, adherence, satisfaction and shorter hospital stay in the intervention group. Zhuang et al. conducted a meta-analysis indicated that digital health interventions with mobile applications (apps), wearable monitors, and web-delivered platforms significantly improved quality of life and self-efficacy (28). However, they found no significant differences in the 6MWD, emergency department admissions, hospital admissions, emergency department or hospital admissions for COPD. IoT-based PR platforms using wearable devices require stronger standardization and quality control. Measured parameters may deviate from reference instruments, so remote monitoring data should be interpreted prudently (29).

### 2.5 Mobile applications

Mobile apps enable smartphone-based data collection and upload, support clinician feedback, and deliver exercise prescriptions and health education to patients. A multicenter RCT found that mobile app-based PR training significantly improved patient prognosis (30). Another multicenter RCT also showed that regular use of smartphone apps helps COPD patients maintain physical activity after PR and achieve sustained improvements in dyspnea and other clinical symptoms (31). Comprehensive mini-program tracking management not only supervises patient exercise and improves adherence but also enables timely collection of medical data for statistical analysis. However, a meta-analysis showed mobile app-based PR yielded better symptoms (lower CAT scores), with no statistic significant in exercise capacity, quality of life and hospitalization outcomes than center-based PR for COPD patients, despite heterogeneous study conditions (32).

### 2.6 Virtual reality

Virtual Reality (VR) creates immersive, interactive simulated scenarios. Combined with artificial intelligence for personalized training, VR-based PR enhances rehabilitation engagement among COPD patients. A meta-analysis demonstrated that VR combined with PR achieved superior outcomes compared with PR alone in improving pulmonary function, alleviating dyspnea symptoms, and increasing 6MWD, alongside improvements in depression and anxiety, suggesting VR may enhance PR effectiveness (33). A comprehensive review indicated that VR shows promising prospects for improving patient prognosis and experience, with strong patient acceptance and practicality. However, further research is required to establish standardized protocols, identify optimal strategies, and evaluate long-term benefits (34). Furthermore, a meta-analysis showed that VR plus conventional therapy produces substantial physical benefits (improved lung function and exercise endurance) and alleviates anxiety and depression, thereby improving quality of life among COPD patients (35). Another meta-analysis indicated that intervention duration affected outcomes; programs lasting 5–12 weeks produced the largest gains in 6MWD. VR-complemented PR was well accepted, with better adherence and engagement than comparator groups (36).

## 3 Application of “Internet Plus” technology in home-based pulmonary rehabilitation for university staff with COPD

Accumulating evidence has verified the general efficacy of “Internet Plus” home-based PR in COPD populations, its translation to specific occupational communities such as university staff remains underexplored, which may represent a logical next step for implementation research. Home-based PR built upon “Internet Plus” technology covers five core modules: rehabilitation guidance, physical exercise training, remote monitoring, health education, and patient self-management. The proposed “hospital-functional community-family” linkage model for home-based pulmonary rehabilitation is depicted in Figure 1.

**Figure 1:**
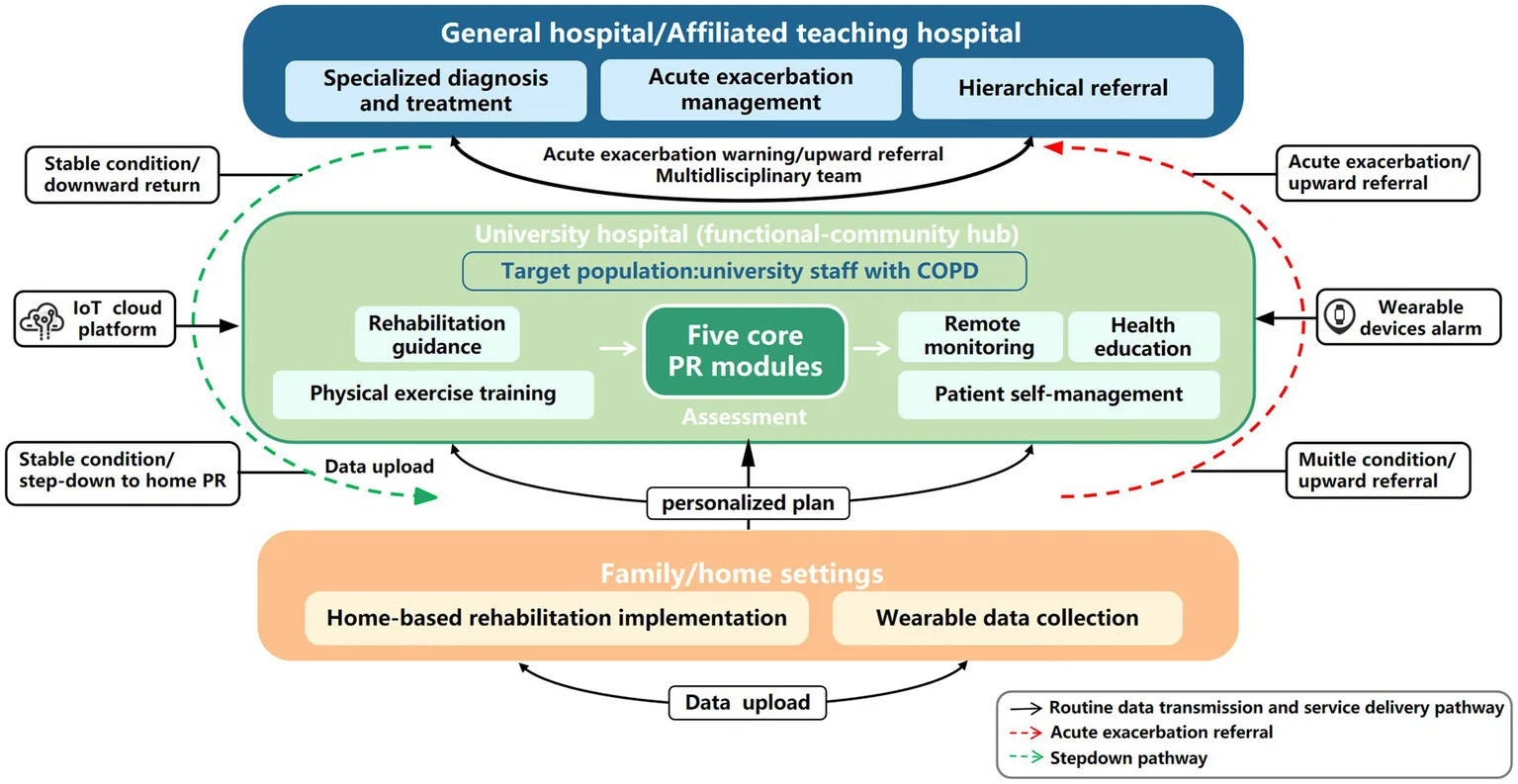
Conceptual framework of the hospital-functional community-family tripartite linkage model for “Internet-Plus” home-based pulmonary rehabilitation for university staff with COPD.

### 3.1 Current status and significance of “Internet Plus” technology in health management among populations of university functional communities

University staff, a typical population in functional communities, face growing risks of chronic and occupational diseases due to work pressure, unhealthy lifestyles and insufficient exercise. Their current health management relies merely on regular physical examinations, lacking closed-loop chronic disease monitoring and targeted primary prevention. The homogeneous characteristics of university communities, combined with “Internet Plus” technologies including telehealth and mHealth, allow staff to utilize fragmented-time health management to optimize chronic disease management, improve clinical outcomes and reduce medical costs (37). Social-media-based online tools facilitate health knowledge access and strengthen patient self-management (38). mHealth has achieved satisfactory effects in hypertension and diabetes management through real-time vital-sign monitoring, improved medication adherence and physical activity, thereby reducing chronic disease complications and improving quality of life. This approach has achieved favorable results in managing hypertension, diabetes, and other chronic diseases. However, relevant research on COPD home-based PR remains fragmented, without standardized intervention usability and evaluation criteria. The integrated use of multiple technologies may yield superior therapeutic effects and warrants clinical promotion. University hospitals, as functional-community service hubs, can build multi-stakeholder collaborative platforms integrating staff physical-examination data and smartphone-app-based lifestyle improvement interventions.

### 3.2 Feasibility of promoting “Internet Plus” technology in home-based pulmonary rehabilitation for university staff with COPD

The high prevalence and low awareness of COPD pose significant challenges for the nationwide implementation of telehealth PR. Current obstacles include a lack of mature technical support and relevant management and supervision systems and low patient participation and adherence. Conducting pilot “Internet Plus” PR within university functional communities helps improve accessibility and efficiency. Notably, all the following advantages are theoretical in nature and have not yet been validated through empirical COPD PR studies within university populations.

#### 3.2.1 Advantages of university staff as a functional community population

Studies have reported COPD among university staff in different countries, with varying prevalence estimates. A survey conducted in China revealed a COPD prevalence of 0.64% among university staff, ranking sixth among prevalent diseases (39). In a 2011 cross-sectional study, researchers identified 11 COPD cases among 685 teachers in Kocaeli, Turkey, through questionnaire surveys, physical examinations, and spirometry, yielding a prevalence of 1.6% (40). Although the absolute number of COPD-affected university staff is small, this population has notable strengths: higher educational attainment, satisfactory digital literacy and strong self-management competence. Geographical concentration enables convenient peer education; low staff turnover and time efficiency guarantees long-term follow-up and data collection, thus enabling rapid comprehension of the operational logic and medical value of “Internet Plus” PR and resulting in high patient adherence. Successful local intervention models can produce spillover demonstrative effects for students and broader communities, thereby forming a demonstrative impact that independently boosts the uptake of “Internet Plus”-enabled PR and cuts promotional expenses.

#### 3.2.2 Resource advantages of university hospitals as functional community facilities

As functional community hospitals, university hospitals are responsible for health management and disease diagnosis and treatment for university staff. Liu et al. developed a self-designed hypertension prevention and management system for the dynamic management of hypertensive patients, which improved patients’ self-management ability and provided a new approach for contracted medical services in functional communities (41). Tang et al. proposed the important role of functional community health service institutions in health management and related promotion strategies (42). As comprehensive primary medical institutions, university hospitals are equipped with basic diagnostic devices including portable spirometers. Supported by affiliated teaching hospitals, they can establish IoT cloud platforms for wearable-device-driven health-data uploading and remote guidance. In addition, multidisciplinary teams can formulate individualized PR regimens. Existing IoT-based chronic-disease management workflows for hypertension and diabetes can be adapted to COPD rehabilitation. Regional responsibility management according to academic departments can be implemented to establish personalized rehabilitation baselines and improve the precision of PR interventions.

#### 3.2.3 Promotion influencing factors and solutions

Major barriers include schedule conflicts, inadequate digital literacy among older adult staff members, insufficient hospital staffing, absence of unified management standards, personal health-data privacy risks, and inadequate funding. Targeted solutions involve peer-health-education, personalized guidance, volunteer follow-up, standardized stratified protocols, cloud-enabled dynamic assessment, strict privacy supervision, and multi-source sustainable funding. “Internet Plus”-based home PR among university staff with COPD are more acceptable than conventional models, with greater benefits at early disease stages. Nevertheless, this digital-first model carries inherent structural limitations: it favors digitally advantaged staff and marginalizes vulnerable subgroups, potentially triggering health inequities and reducing generalizability. Future studies should develop stratified, hybrid PR combining digital and offline support to reduce inequity and improve generalizability among university staff.

## 4 Discussion

This mini review summarizes the application of “Internet Plus” technology in home-based PR for COPD patients, and explores its potential translational value for university staff populations. As a novel home-based PR model, “Internet Plus”-enabled PR shows promising prospects for long-term COPD management. Nevertheless, existing studies have yielded inconsistent findings regarding remote PR. An umbrella review on COPD exacerbation prediction and management showed that artificial intelligence improves exacerbation prediction and that diverse digital interventions optimize whole-course COPD care to reduce exacerbations (43). Another umbrella review reported that digital self-management interventions benefited physical activity, quality of life, lung function, adherence and mood, but showed inconclusive effects on dyspnea, physical capacity, sedentary time, emergency visits and mortality (44). However, a recent meta-analysis found no statistically significant differences in functional capacity, dyspnea or health-related quality of life between tele-rehabilitation and conventional center-based PR (45). Such discrepancies arise from methodological heterogeneity across primary studies. Variable intervention duration and intensity produce inconsistent functional benefits. Differences in outcome definitions and follow-up timepoints lead to conflicting results. Inconsistent control conditions, ranging from usual care to center-based PR, attenuate intervention effects. Variations in participant age, COPD severity, and enrolment status at stable or post-exacerbation stages further contribute to inconsistent findings. Collectively, these factors make remote digital PR a non-uniform intervention and explain the inconsistent efficacy observed in prior syntheses. In terms of specific intervention strategies, a network meta-analysis involving seven intervention measures showed positive results (46). The combination of smart wearable devices and telephone support, as well as smart wearable devices alone, could improve patients’ physical activity levels; interventions based on the WeChat platform, Apps, and smart wearable devices effectively enhanced patients’ exercise endurance. We propose that evidence from general COPD cohorts is relevant to university staff with COPD. Barriers including sedentary work, heavy workloads and limited spare time make this group theoretically suitable for “Internet Plus” home-based PR. Nevertheless, remote PR for COPD patients in university functional communities lacks published empirical evidence, and this remains a conceptual translational argument. “Internet Plus”-based remote PR offers high accessibility and flexibility, which may effectively address the problems of insufficient medical resources and poor patient compliance that beset conventional COPD rehabilitation. This potential advantage is particularly relevant for university staff with sub-health conditions, for whom wearable-assisted home rehabilitation could be more targeted and operable. The high educational level and digital literacy of university staff, together with the multidisciplinary resources of university hospitals, might facilitate rapid replication and promotion of this model. However, improvements in technical stability, cost-effectiveness, and applicability are still required. A recent review highlighted concerns over the long-term operational costs of wearables and digital platforms, with inadequate high-quality evidence confirming their sustained cost-effectiveness (47). Economic findings for digital interventions remain conflicting, with most studies offering only partial cost estimates rather than long-term economic evaluations. Telerehabilitation models will need tailoring to their locally available resources and the costs individualized to their respective healthcare environments (48). Digital health barriers exist at system, healthcare provider and patient levels, and these multifaceted challenges impede the uptake of Internet Plus home-based PR for COPD. Within China, policy constraints add further complexity: the Personal Information Protection Law governs health data collection and transmission, imposing strict data governance requirements on IoT and cloud rehabilitation platforms (49). Meanwhile, limited reimbursement for telehealth PR undermines sustainable funding for such digital programs. Future research should fill this critical evidence gap by conducting prospective, hypothesis-driven pilot studies targeting university staff with COPD to establish standardized “Internet Plus” PR management protocols and data governance frameworks for university settings. Additional work should develop age-friendly interfaces to reduce technical barriers, improve long-term follow-up and cost-effectiveness assessments, and evaluate real-world acceptability and clinical outcomes before widespread implementation of “Internet Plus” home-based PR in universities. Ultimately, building a “hospital-functional community-family” collaborative linkage involving general hospitals, university hospitals and families could enable early detection of acute exacerbations, tiered referral and closed-loop long-term follow-up, thereby supporting full-cycle COPD management.
