# Supplementary material for: The value of promoting “Internet Plus” home-based pulmonary rehabilitation for university staff with chronic obstructive pulmonary disease
Source: Front Public Health. 2026 Sep 30;14:1976082. doi: 10.3389/fpubh.2026.1976082 (PMC13662792; doi:10.3389/fpubh.2026.1976082)
Supplement: Supplementary file 1 [file Data_Sheet_1.DOCX]

**Search strategy**

A comprehensive search was conducted in PubMed, CNKI and Wanfang databases for publications from January 2015 to August 2026, using the keywords of “Internet plus”, “digital health”, “chronic obstructive pulmonary disease (COPD)”, “university staff”, “university faculty”, “home-based respiratory rehabilitation” and “telerehabilitation”.

**PubMed search string :**("chronic obstructive pulmonary disease"[Title/Abstract] OR COPD[Title/Abstract]) AND ("telemedicine"[Title/Abstract] OR telerehabilitation[Title/Abstract] OR "home-based rehabilitation"[Title/Abstract] OR "home respiratory rehabilitation"[Title/Abstract] OR "digital health"[Title/Abstract] OR "Internet plus"[Title/Abstract])

**CNKI and Wanfang search strings:** (慢性阻塞性肺疾病 OR COPD) AND (远程康复 OR 居家呼吸康复 OR 数字健康 OR 互联网+).

**Inclusion criteria:** (1) participants diagnosed with chronic obstructive pulmonary disease; (2) the intervention was Internet plus, telerehabilitation or home-based respiratory rehabilitation; (3) they were peer-reviewed research articles, systematic reviews, or meta-analysis, or reports from the WHO; (4) articles written in Chinese or English.

**Exclusion criteria:** (1) duplicate publications; (2) case reports, conference abstracts, letters, animal or cell laboratory studies; (3) publications without accessible full text; (4) articles in languages other than Chinese or English.

Duplicates across the three databases were manually removed using EndNote. Records in languages other than English or Chinese were excluded at the title/abstract screening stage.

All retrieved publications were screened regardless of whether the study population comprised university staff/faculty. For the screening flow, 1315 records were initially retrieved from the three databases. After deduplication, 563 unique records remained. Title and abstract screening excluded 452 irrelevant records, leaving 111 articles for full-text assessment. Full-text screening further excluded 61 ineligible articles, and 41 studies were included in original form of this mini review. Additional literature was retrieved later according to the comments from reviewers and editors, and 49 studies were finally included.
